# Generation of segmental chips in metal cutting modeled with the PFEM

**DOI:** 10.1007/s00466-017-1442-z

**Published:** 2017-09-01

**Authors:** J. M. Rodriguez Prieto, J. M. Carbonell, J. C. Cante, J. Oliver, P. Jonsén

**Affiliations:** 1 Division of Mechanics of Solid Materials, Luleå University of Technology (LTU), Luleå, Sweden; 2 Escola Tècnica Superior d’Enginyeries Industrial i Aeronàutica de Terrassa, Rambla de Sant Nebridi 22, 08222 Terrassa, Spain; 3 E.T.S dEnginyers de Camins, Canals i Ports, Technical University of Catalonia (BarcelonaTech), Campus Nord UPC, Mòdul C-1, c/ Jordi Girona 1-3, 08034 Barcelona, Spain; 4 International Center for Numerical Methods in Engineering (CIMNE), Campus Nord UPC, Gran Capitán, s/n., 08034 Barcelona, Spain

**Keywords:** Particle Finite Element Method (PFEM), Metal cutting processes, Serrated chip formation

## Abstract

The Particle Finite Element Method, a lagrangian finite element method based on a continuous *Delaunay* re-triangulation of the domain, is used to study machining of Ti6Al4V. In this work the method is revised and applied to study the influence of the cutting speed on the cutting force and the chip formation process. A parametric methodology for the detection and treatment of the rigid tool contact is presented. The adaptive insertion and removal of particles are developed and employed in order to sidestep the difficulties associated with mesh distortion, shear localization as well as for resolving the fine-scale features of the solution. The performance of PFEM is studied with a set of different two-dimensional orthogonal cutting tests. It is shown that, despite its Lagrangian nature, the proposed combined finite element-particle method is well suited for large deformation metal cutting problems with continuous chip and serrated chip formation.

## Introduction

The development of a new machining process requires considerable investment of time and resources. Precise knowledge of the optimal cutting conditions is essential for a timely start-up. Process characteristics such as the tool geometry and cutting speed directly influence chip morphology, cutting forces, final product dimensionality and tool life. Computer simulations of the cutting processes can potentially reduce the number of design iterations and result in substantial cost savings.

The study of metal cutting is difficult from an experimental point of view, experiments are difficult to carry out. Among the reasons there are the high speed at which the cut takes place under industrial machining conditions and the small scale of the phenomena which are to be observed. Furthermore, the continuous development of tool and work piece materials and the continuous development of tool geometries makes the testing a laborious and hard task.

The chip formation process in cutting is difficult to analyze using analytical methods, due to that, finite element modeling (FEM) has frequently been used to study the process of chip formation at high cutting speeds, see for example [12, 18, 25, 31]. Numerical modeling of machining processes is continuously attracting researchers for better understanding of chip formation processes, the heat generation in cutting zone, tool-chip inter facial frictional characteristics and quality of the machined surfaces. Finite element simulations allow studying the cutting process in greater detail than possible in experiments. In the modeling of machining processes, the workpiece material is highly deformed due to the cutting. In a *Lagrangian* analysis, this deformation produces a large mesh distortion. Element distortion has been always matter of concern which limited the analysis to incipient chip formation in some studies. Standard *Lagrangian* approaches such as FEM cannot resolve the large deformations very well. Instead, FEM with an *Eulerian* formulation requires the knowledge of the chip geometry in advance and the mesh is fixed in the space, that, undoubtedly, restricts the range of cutting conditions capable of being analyzed. Therefore, modeling metal cutting processes becomes a major challenge for the finite element method (FEM).

The main demanding task is related with the modeling and simulation of large configuration changes. Numerical simulations of cutting process involves angular distortions and large strains, generation of new boundaries, multiple contacts and self-contact, and fracture with multiple cracks. All of them are difficult to manage using standard FEM. The main objective of this paper is specifically to contribute to the solution of some of the problems described above through the application of the Particle Finite Element Method (PFEM) to the solution of metal cutting processes including the generation of continuous but also segmented chips.

### Contents

The paper starts with the description of the problem (see Sect. 2), explaining which elements come into play in the numerical simulation and complex physical phenomena occur which during chip formation.

In Sect. 3 the coupled thermo-mechanical problem equations are presented in a summarized form of the initial boundary value problem (IBVP). The equations are written using the weak form of the problem and particularized with the mixed displacement-pressure formulation which prompted the use of stabilization.

In Sect. 4 we describe the contact phenomena at the workpiece-tool interface with a rigid tool contact. In Sect. 5 we present an overview of the thermo-elastoplastic models at finite strains and its stress-update algorithm.

Details of the implicit solution of the Lagrangian FEM equations in time using an updated Lagrangian approach and a Newton-Raphson iterative scheme are presented in Sect. 6.

Then, in Sect. 7, the basic general steps of the PFEM and the custom characteristics of the present formulation are explained.

From the model explained in Sect. 5.2 a set of examples are analyzed in Sect. 8 in order to test the capabilities of the present formulation for predicting the transition from continuous to serrated chip formation when an increase of the cutting speed is applied.

## Problem statement

In this section, the complex physical phenomena that occur during chip formation and the numerical model developed within this work is described.

### Description of the physical problem

High speed machining has a lot of advantages in comparison with traditional machining. The productivity can be improved by increasing material removal rate, decreasing cutting forces and enhancing surface quality. Complex physical phenomena occur during chip formation, such as adiabatic shearing bands formation. Thermal softening of the material in the shear zone leads to an increase of the deformation in this zone, which produces heat and leads to further softening. This positive feedback between softening and deformation causes a narrow band (adiabatic shear band) of extremely large deformation, while the surrounding material is only slightly deformed. For this process to be plausible, the thermal softening must be larger than strain and strain rate hardening, so that the effective stress-strain curve of a material point inside the shear localized region has a maximum. The cutting speed must be high enough so that heat conduction out of the shear zone is small or negligible.

In this work we present the segmental chip formation, which segmentation is solely caused by the described mechanism of adiabatic shearing. In many applications, machining is one of the main cost-determining factors, especially when hard-to-machine alloys like the titanium alloy Ti6Al4V have to be cut. Due to the fact that titanium is a poor thermal conductor, titanium alloys form segmented chips, where the deformation of the chip is inhomogeneous and regions of large and small deformation alternate, leading to a serrated back side of the chip. Large deformations and changes in geometry occurring during chip formation and segmentation make the numerical simulations of machining a big challenge. The main objective of this work is precisely to contribute to solve some of the problems described above through the use of the Particle Finite Element Method (PFEM) and its main ingredients: (1) continuous Delaunay triangulation and (2) insertion and removal of particles.

### Description of the numerical model

**Fig. 1:**
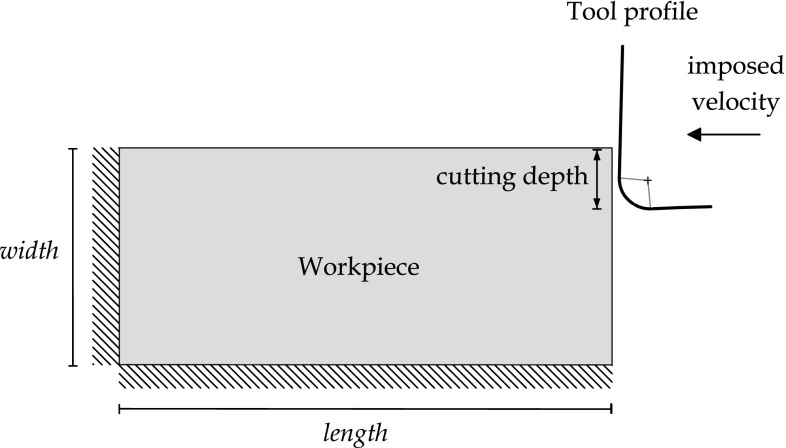
Linear cutting test model

The model of unsteady chip formation that will be analyzed in this work is shown in Fig. 1. It represents a linear cutting test with some particular characteristics. To simplify the process complexity, the tool is considered rigid and will be defined in a parametric way, having a rake angle, a flank angle and a tool radius. The process is studied using a reduced two-dimensional model, which strongly improves the computational times and gives a clearer representation of the resultant thermo-mechanical response. The cutting tool is moved at a constant speed from the right to the left. The width of the cutting edge is larger than that of the work piece and it extends on both sides of the work piece. The work piece material is considered as homogeneous, isotropic and initially unstressed. Also, the work piece acceleration and the induced anisotropy are neglected. The motion of the left and lower parts of the work piece are restricted in the horizontal and vertical directions. Cutting begins at time $t=0$. Build up of the workpiece on the tool and subsequent growth of the chip is to be simulated in real time. The model accounts for heat conduction, heat generation through plastic work dissipation and workpiece-tool contact neglecting friction. The modeling used in this work is able to predict the transition between continuous and serrated chip formation when increasing the cutting speed, this happens due to adiabatic shearing experienced by the material. Material separation in front of the tool has been modeled by considering the chip formation process as pure deformation where material flows visco-plastically around the tool tip.

## Equation of motion, thermal effects and balance of mass

A solid domain containing a deformable material which evolves in time due to the external and internal forces is considered. Displacements and thermal conditions from an initial configuration at time $t=0$ to a current configuration at time $t=t_n$ are prescribed in some parts of the present domain. The volume *V* and its boundaries $\Gamma$ at the initial and current configurations are denoted as $(V_0,{}\Gamma _{0})$ and $(V_n,\Gamma _{n})$, respectively. The aim is to find the spatial configuration that the material is going to occupy at time $t=t_n+\Delta t$, and, at the same time, obtain displacements, strains, and stresses in the updated configuration. In the present work, we will choose simple 3-noded linear triangle to discretize the displacement, the pressure and the temperature fields (PFEM requirement). Higher order elements can also be used, see [32].

### Balance of momentum

The Balance of momentum is enforced weakly at time $t=t_{n+1}$ by recourse to the virtual work principle

1 $$\begin{aligned}&\int _{V_t}{\delta {{\varepsilon }_{ij}}\,{{s}_{ij}}}\,d{V_t}+ \int _{V_t}{\delta {{\varepsilon }_{ij}}\,p\,{{\delta }_{ij}}}\,d{V_t}- \int _{V_t}{{{w}_{i}}\,{{b}_{i}}}\,d{V_t}\nonumber \\&\quad - \int _{\Gamma _{\sigma }}{{{w}_{i}}\,t_{i}^{p}}d\Gamma _{\sigma } = 0 \end{aligned}$$

where $V_t$ is the volume occupied by the solid in the current configuration, ${{s}_{ij}}$ is the deviatoric part of the Cauchy stress tensor and *p* is the pressure, ${b}_{i}$ are the external body forces and $t_{i}^{p}$ the prescribed surface forces, ${\delta {\varepsilon }_{ij}}$ is a virtual strain field and ${w}_{i}$ are the space weighting functions for the displacement field.

Upon discretization of (1) with finite elements the governing equations become

2 $$\begin{aligned} {{\mathbf {F}}_{\mathbf {u},{\text {int}}}}(\mathbf {u},p)-{{\mathbf {F}}_{ext}}=0 \end{aligned}$$

where

3 $$\begin{aligned}&\mathbf {F}_{\mathbf {u},int}(\mathbf {u},\mathbf {p}) = \int _{V_t}{\mathbf {B}_u}^T{( {{{\varvec{s}}}}(\mathbf {u}) + p})\,d{V_t} \end{aligned}$$

4 $$\begin{aligned}&\mathbf {F}_{\mathbf {u},ext} = \int _{V_t}{\mathbf {N}^T\mathbf {b}}\,d{V_t} - \int _{\Gamma _{\sigma }}{\mathbf {N}}\mathbf {t}^{p}\,d{\Gamma _{\sigma }} \end{aligned}$$

where $\mathbf {N}$ are the global shape functions and $\mathbf {B}_u$ is the strain-displacement matrix. The $\mathbf {B}_u$ matrix contains the derivatives of the shape functions used in the interpolation of the problem variables.

### Thermal balance

In applications such as metal cutting, substantial amount of heat may be generated due to the plastic working of the solid. Temperatures attained can be quite high and have a considerable influence on the mechanical response. The relevant balance law, in this case, is the first law which can be expressed in weak form as

5 $$\begin{aligned}&\int _{V_t}{\hat{w}\rho \,c\,\dfrac{d\theta }{dt}}\,d{V_t}+ \int _{V_t}{\frac{\partial \hat{w}}{\partial {{x}_{i}}}(k\,\dfrac{\partial \theta }{\partial {{x}_{i}}})\,d{V_t}}+ \int _{V_t}{\hat{w}\,Q\,d{V_t}}\nonumber \\&\quad + \int _{\Gamma _{\mathbf {q}}}{\hat{w} q_{n}^{p}}\,d\Gamma _{\mathbf {q}} = 0 \end{aligned}$$

where $\theta$ is the temperature, $\rho$ is the density, and *c* the specific heat, and *k* the thermal conductivity. *Q* is the thermal source and $q_{n}^{p}$ the flux in the boundary. $\hat{w}$ are the space weighting functions for the temperature. The rate of heat supply due to the plastic deformation in the bulk is estimated as

6 $$\begin{aligned} Q=\beta {{\dot{W}}^{p}} \end{aligned}$$

where $\dot{W}^{p}$ is the plastic power per unite of deformed volume and $\beta$ the Taylor-Quinney coefficient. Inserting the finite element interpolation into (5) results in the semi-discrete system of equations

7 $$\begin{aligned} \mathbf {F}_{\theta ,dyn}(\dot{\theta })-\mathbf {F}_{\theta ,int}(\theta ) +\mathbf {F}_{\theta ,ext}=0 \end{aligned}$$

where

8 $$\begin{aligned}&\mathbf {F}_{\theta ,int}(\theta ) = \int _{V_t} k{\mathbf {B}_{\theta }}^T{\mathbf {B}_{\theta }}\,d{V_t} - \int _{V_t}\mathbf {N}^T Q\,d{V_t} \end{aligned}$$

9 $$\begin{aligned}&\mathbf {F}_{\theta ,ext} = \int _{\Gamma _{\mathbf {q}}}\mathbf {N}^T(\mathbf {q}^{p}\cdot \mathbf {n})\,d{\Gamma _{\mathbf {q}}} \end{aligned}$$

10 $$\begin{aligned}&\mathbf {F}_{\theta ,dyn}(\dot{\theta }) = \int _{V_t}{{\rho }c\,\mathbf {N} \mathbf {N}^T}\dot{\theta }\,d{V_t} \end{aligned}$$

where $\mathbf {B}_{\theta }$ is the global gradient-temperature matrix respectively. The $\mathbf {B}_{\theta }$ matrix contains the derivatives of the shape functions used in the interpolation of the temperature field.

A Backward Euler method is used to discretize (7) in time, with the result

11 $$\begin{aligned} \mathbf {F}_{\theta ,dyn}({\dot{\theta }}_{n+1})-\mathbf {F}_{\theta ,int} (\theta _{n+1})+\mathbf {F}_{\theta ,ext}=0 \end{aligned}$$

where

12 $$\begin{aligned} {\dot{\theta }}_{n+1}=\dfrac{\theta _{n+1}-\theta _{n}}{\Delta t} \end{aligned}$$

and $\Delta t$ is the size the length of the time interval.

### Mass balance

The mass conservation equation can be expressed in weak form as

13 $$\begin{aligned} \int _{V_t}{-\dfrac{q}{\kappa }\,p}\,d{V_t} + \int _{V_t} q \left( \ln (J)-3\,\alpha \,\dfrac{(1-\ln (J))}{J}(\theta -\theta _0)\right) \,d{V_t}\ =0 \end{aligned}$$

where *J* is the determinant of the deformation gradient, $\alpha$ is the thermal expansion coefficient, $\theta _0$ is the temperature of the solid at the beginning of the cutting processes and $\kappa$ is the incompressibility modulus. *q* represents the space weighting functions for the pressure.

Upon discretization with finite elements, the incompressibility balance equations (13) are written as

14 $$\begin{aligned} \mathbf {F}_{\mathbf {p},pres}(\mathbf {p})-\mathbf {F}_{\mathbf {p},vol}(\mathbf {u})=0 \end{aligned}$$

where

15 $$\begin{aligned}&\mathbf {F}_{\mathbf {p},pres}(\mathbf {p}) = \int _{V_t}\dfrac{1}{\kappa }\mathbf {N}\mathbf {N}^T p\,d{V_t} \end{aligned}$$

16 $$\begin{aligned}&\mathbf {F}_{\mathbf {p},vol}(\mathbf {u}) = \int _{V_t} \mathbf {N}^T \left( \ln (J)-3\,\alpha \,\dfrac{(1-\ln (J))}{J}(\theta -\theta _0)\right) \,d{V_t}\nonumber \\ \end{aligned}$$

In finite element computations, the force vectors presented in Eqs. 2, 11 and 14, are obtained as the assemblies of element vectors. In this work, the element force vectors are evaluated using Gaussian quadratures.

#### Stabilization of the mixed displacement-pressure equation

The selected discretization (linear elements for displacement and pressure) is known to lead to spurious oscillations in the pressure field and a stabilization procedure is required. For this purpose, the stabilized formulation called the Polynomial Pressure Projection (PPP) presented and applied to the Stokes equations in [8] is used. The PPP introduces a pressure term in the mass conservation equation. Unlike other stabilization methods, the Polynomial pressure projection (PPP) does not require specification of a mesh dependent stabilization parameter or calculation of higher-order derivatives. The PPP uses a projection on a discontinuous space and as a consequence can be implemented in an elementary level surpassing the need of mesh dependent and problem dependent parameters. Using the PPP stabilization strategy, the balance of mass is re-written as

17 $$\begin{aligned} \mathbf {F}_{\mathbf {p},pres}(\mathbf {p})-\mathbf {F}_{\mathbf {p},vol} (\mathbf {u})+\mathbf {F}_{\mathbf {p},stab}(\mathbf {p})=0 \end{aligned}$$

where

18 $$\begin{aligned} \mathbf {F}_{p,stab}(\mathbf {p}) = \int _{{V_t}^{(e)}}\dfrac{\alpha _s}{\mu }\,\mathbf {p}^{(e)}\left( {\mathbf {N}}^{(e)} {\mathbf {N}^{T\,(e)}}-{\tilde{\mathbf {N}}}^{(e)}{\tilde{\mathbf {N}}^{T\,(e)}}\right) \,d{{V_t}^{(e)}} \end{aligned}$$

and ${\tilde{\mathbf {N}}}^{(e)}$ contain the set of polynomials of order 0, $\mu$ is the shear modulus and $\alpha _s$ a parameter that takes the value 1. More details about the implementation of the PPP strategy are found in [19–22].

**Fig. 2:**
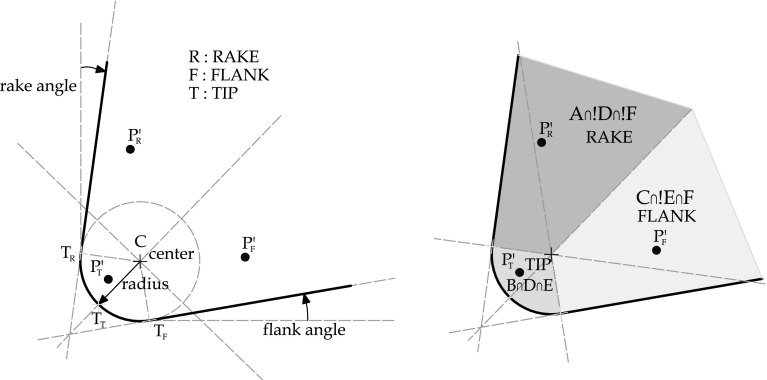
Rigid tool definition parameters and contact zones. Contact zones defined by the intersection of the groups depicted in Fig. 3

## Contact

### Rigid tool contact

The approach followed in this work to take into account the interaction between a cutter and a workpiece is to consider the tool as a rigid element and the workpiece as deformable body. If the cutter is rigid, the geometry of that element is not going to change during the contact. A simple way to define a fixed geometry is using the mathematical description of its boundary. That allows us to avoid the drawbacks coming from the treatment of contact between finite elements or a boundary with described by a mesh of finite elements.

When contact between finite elements is considered, the mechanics turns to something numerically more complex. A geometrical contact search is required and a special master-slave structure is needed to solver the contact interaction. If the tool surface is discretized with standard finite elements, there is no continuity from one element to another. Therefore the discrete interface gives a non-smooth contact force profile. The lack of continuity degrades the convergence of the solution.

In order to obtain a good definition of the contact forces a good characterization of the contacting geometry is needed. This is done using a parametrization of the tool surface. Once an active set of contacts are determined, the mathematical description of the contact constraint is included in the linear momentum balance equation.

#### Parametrization of the contact tool surface

**Fig. 3:**
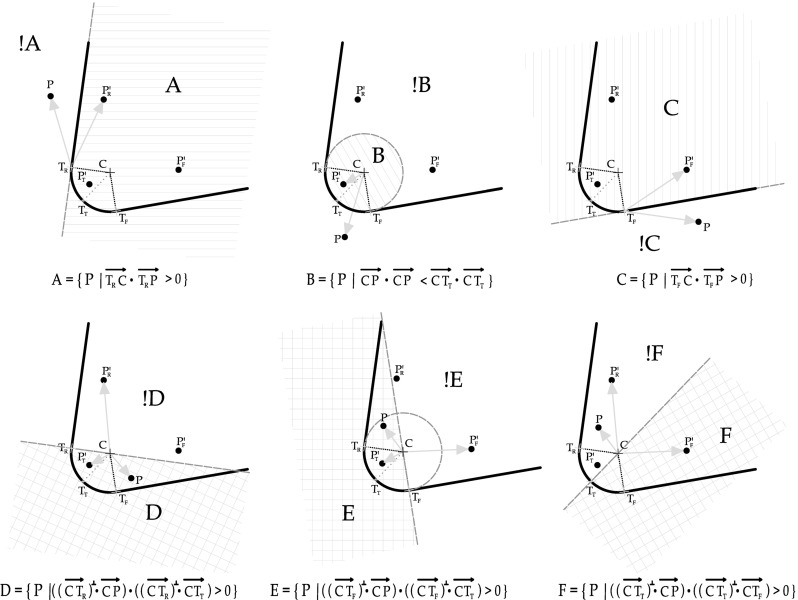
Geometrical contact search based on the definition of spatial rigid tool contact zones

The geometrical scheme considered to describe a cutting tool can be characterized with three parameters: the tip radius, the angle of the rake face (rake angle) and the angle of the flank face (flank angle) (see Fig. 2). Defining these three parameters a circle (or a cylinder in 3D) and two planes tangent to that circle are mathematically determined. For the contact detection, one must also define the exterior side and the interior size of the tool. When a particle of the deformable domain is going to exceed the described contour that particle will be in contact with the tool. The geometrical contact definition is performed by any particle of the workpiece boundary. The contact parameters will be defined by the contact zone where a particle is going to interact with. Taking into account the characteristics of the parametrization, these zones can be classified as the rake, the tip and the flank side of the cutting tool (see Fig. 2). The spatial zones are determined by the intersection of geometrical areas defined by the subgroups shown in Fig. 3. Each subgroup is defined geometrically using the projection of auxiliary vectors coming from the geometrical characterization of the tool parametrization.

#### Normal contact contribution

The definition of the outward normal direction $\mathbf {n}$ will be given by the mathematical equation characterizing the rigid tool surface. A boundary workpiece particle contacting to the rake of flank areas will got the normal of that planes. In the case of a particle contacting with the tip contour the normal will be characterized by the segment joining the particle with the tool center. Once a particle enters in contact with one of the tool surfaces the normal gap $g_N$ will be computed projecting the exceeding distance vector into the corresponding normal. Using a penalty approach, the constitutive equation for the normal force $P_N$ is given by

19 $$\begin{aligned} {P_N}=\kappa {g_N} \end{aligned}$$

where the $\kappa$ is the penalty parameter. Note that $\kappa$ must be proportional to the contributive surface area of the particle entering in contact in order to ensure that the finite elements pass the patch test. The contact constraint contribution for the weak form of the linear momentum equation is given by

20 $$\begin{aligned} \int _{\Gamma _{cont}}{w}_{i}\,P_N\,n_{i}\,d\Gamma _{cont} = P_N \mathbf {n} \end{aligned}$$

$\mathbf {n}$ is the normal to the contact surface.

## Constitutive model and stress-update algorithm

### Thermo-elastoplasticity model at finite strains

In this section, the formulation of the constitutive thermo-plasticity model at finite strains will be summarized. In Box 1 we present the main equations of the thermo-mechanical $J_2$ flow model for rate dependent plasticity that will be used in this work. Details of the theory of thermo-plasticity as well the definition of the variables that appear in Box 1 are explained see [19–21, 29]. In this model, we assume that the deformation gradient $\mathbf {F}$ admit a multiplicative decomposition in elastic and plastic parts, $\mathbf {F} =\mathbf {F^e}\mathbf {F^p}$, respectively. The elastic response is expressed in terms of $\mathbf {b^e} = \mathbf {F^e} \mathbf {F^e}^{T}$, where $\mathbf {F^e}$ is the elastic left Cauchy-Green deformation tensor. The elastic response of the material is modeled using a hyperelastic Neo-Hookean model [3, 27].

The implicit Backward-Euler method is the most commonly used integration scheme for plasticity (see [27, 28, 30]) and it is the integration scheme adopted in this work. Other integration schemes are available in the literature see [13, 15, 21]. Box 2 shows the main steps involved in the radial return mapping algorithm to solve the $J_2$ plasticity model presented in Box 1.

**Table 1:** Coupled thermo-mechanical $J_2$ flow theory. Rate dependent plasticity

1.	Free energy function.
	$$\hat{\psi } = \hat{T}(\theta ) + \hat{M}(\theta ,J^e)+\hat{U}(J^e)+\hat{W}(\bar{\mathbf {b}}^e)+\hat{K}({\bar{e}\,}^p,\theta )$$
2.	kirchhoff stress.
	$$\begin{aligned} {{{\varvec{\tau }}}}&= J^e\,p\,\mathbb {1}+\mathbf {s}\\ p&:= \left[ -3\,\alpha \,\kappa \,\dfrac{(1-\ln (J^e))}{J^e}(\theta -\theta _0)+ \kappa \,\ln (J^e)\right] \\ \mathbf {s}&:= \mu \,{ dev}(\bar{\mathbf {b}}^e) \\ \mathrm {and\;the\;entropy}&\\ \eta&= \eta ^p - \eta ^e + \eta ^t \\ \eta ^e&:= - \partial _{\theta }\hat{T}(\theta )\\ \eta ^t&:=-\partial _{\theta }\hat{M}(\theta ,J^e)-\partial _{\theta }\hat{K}({\bar{e}\,}^p,\theta ) \end{aligned}$$
3.	*Von Mises* yield criterion.
	$$\Phi (\tau ,{\bar{e}\,}^p,\theta )=\Vert dev (\tau )\Vert -\sqrt{\dfrac{2}{3}}\left( \sigma _y+\beta \right) \left( {\dot{\bar{e}}^p} \right) \le 0$$
4.	Evolution equations $\lambda >0,\;\;\Phi \le 0,\;\;\lambda \,\Phi =0$
	$$\begin{aligned} \mathcal {L}_v\mathbf {b}^e= & {} -2\,\lambda \,J^{-\frac{2}{3}}\dfrac{1}{3}{} tr (\bar{\mathbf {b}}^e)\mathbf {n}\\ {\dot{\bar{e}}^p}= & {} -\lambda \,\partial _{\beta }\Phi (\tau ,{\bar{e}\,}^p,\theta )\\ {\dot{\eta }}^p= & {} \lambda \,\partial _{\theta }\Phi (\tau ,{\bar{e}\,}^p,\theta ) \end{aligned}$$
The definition of the variables that appear in this box are explained in [21, 27, 29].

**Table 2:** Implicit Backward-Euler integration flowchart for thermo-elastoplasticmodels

1.	Thermoelastic trial state:
	Initial data: $\;\bar{\mathbf {b}}^e_n,\,{\bar{e}\,}^p_n,\,\eta ^p_n$
	Current values of $\mathbf {F}_{n,n+1},\, \theta _{n+1}$, where $\;\bar{\mathbf{F}}_{n,n+1}=J^{-\frac{1}{3}}\mathbf {F}_{n,n+1}$
	Let $\;\;f^{trial}_{n+1} = \left\| \mathbf {s}^{trial}_{n+1}\right\| - \sqrt{\dfrac{2}{3}}\left( \sigma _{y,n+1}+\beta _{n+1}({\bar{e}}^{\,p}_n)\right)$
	IF $f^{trial}_{n+1}\le 0\,$: Set $\;\;(\bar{\mathbf {b}}^e_{n+1},\,{\bar{e}\,}^p_{n+1},\,\eta ^p_{n+1}) = (\bar{\mathbf {b}}^{e,trial}_n,\,{\bar{e}\,}^p_n,\,\eta ^p_n\;\;)\;$ and EXIT
	ELSE:
2.	Consistency parameter:
	Set $\bar{\mu }= \dfrac{\mu }{3}{} tr (\bar{\mathbf {b}}^{e,trial}_{n+1})$
	Compute $\Delta \lambda _{n+1}$ by solving:
	$$\begin{aligned} g(\Delta \lambda _{n+1}) =&f^{trial}_{n+1} - 2\,\Delta \lambda _{n+1}\,\mu \,\dfrac{1}{3} tr (\bar{\mathbf {b}}^{e,trial}_{n+1})\\ +&\sqrt{\dfrac{2}{3}}\left( \sigma _{y,n}+\beta _{n}({\bar{e}}^{\,p}_n)\right) - \sqrt{\dfrac{2}{3}}\left( \sigma _{y,n+1}+\beta _{n+1}({\bar{e}}^{\,p}_{n+1})\right) = 0 \end{aligned}$$
	Return mapping:
	Set $\mathbf {n}_{n+1} = \dfrac{\mathbf {s}^{trial}_{n+1}}{\Vert \mathbf {s}^{trial}_{n+1}\Vert }$ and update
	$$\begin{aligned} \mathbf {s}_{n+1} =&\mathbf {s}^{trial}_{n+1} - 2\,\Delta \lambda _{n+1}\,\mu \,\dfrac{1}{3} { tr}(\bar{\mathbf {b}}^{e,trial}_{n+1})\mathbf {n}_{n+1}\\ {\bar{e}\,}^p_{n+1} =&{\bar{e}\,}^p_n - \lambda _{n+1}\Delta t\sqrt{\dfrac{2}{3}}\\ \eta ^p_{n+1} =&\eta ^p_n - \sqrt{\dfrac{2}{3}}\Delta \lambda _{n+1} \partial _{\theta } \left( \sigma _{y,n+1}+\beta _{n+1}({\bar{e}}^{\,p}_{n+1})\right) \end{aligned}$$
3.	Update the intermediate configuration by the closed form formula:
	$$\bar{\mathbf {b}}^e_{n+1} = \bar{\mathbf {b}}^{e,trial}_{n+1} - 2\,\Delta \lambda _{n+1}\,\dfrac{1}{3}{} { tr}(\bar{\mathbf {b}}^{e,trial}_{n+1})\mathbf {n}_{n+1}$$
	END
The definition of the variables that appear in this box are explained in [21, 27, 29].	

### Isotropic Hardening law

In a typical machining event, very high strain rates in excess of $1\cdot 10^{7}\,s^{-1}$ may be attained within the primary shear zone, while the remainder of the workpiece deforms at moderate or low strain rates. A simple model which accounts for this behavior is described in the following lines.

As it is the intent of this paper to show that PFEM is able to predict the main effects of the cutting speed on chip formation, a rather simple, generic flow stress law has been used as is described in the material model presented by *Bäker* in [1]. The isothermal flow stress $(\sigma _y+\beta )$ used in this paper is given by

21 $$\begin{aligned} \left( \sigma _y+\beta \right) \left( {\bar{e}}^{p},{\dot{\bar{e}}}^{p},\theta \right) =K(\theta ){{({{\bar{e}}^{p}})}^{n}}\left( 1+C\ln \left( \frac{{\dot{\bar{e}}}^{p}}{{\dot{\bar{e}}}_{0}}\right) \right) \end{aligned}$$

where *K* and *n* are temperature-dependent material parameters, ${\dot{\bar{e}}}_{0}$ the reference strain rate and *C* is a constant. More detail can be found in [1].

The temperature dependence of the parameters has the form:

22 $$\begin{aligned} K(\theta )= & {} {{K}^{*}}\Psi (\theta ) \;\;\;\;\;\; n(\theta )={{n}^{*}}\Psi (\theta ) \nonumber \\ \Psi (\theta )= & {} exp\left( -\left( \frac{\theta }{\theta _{MT}}\right) ^{\mu }\right) \end{aligned}$$

$K^*$, $n^*$, $\theta _{MT}$ and $\mu$ are material properties see([1]). Values for these parameters and thermophysical data are listed in Tables 1 and 2. It should be noted that this flow stress law should only be considered as an approximation to the real material due to the large extrapolations necessary. The extrapolation is necessary because the split-Hopkinson bar apparatus allows to characterize the material at strain rates of up to $1\cdot 10^{4}\,s^{-1}$ at different temperatures but in high speed machining, the strain rates over $1\cdot 10^{7}\,s^{-1}$ are reached.

**Table 3:** Mechanical properties titanium alloy Ti6Al4V for the *Bäker* model

*C*	0.302	
${\dot{\bar{e}}}_{0}$	774	$\hbox {s}^-1$
$K^*$	2260	${\mathrm {MPa}}$
$n^*$	0.339	
$\theta _{MT}$	825	$\mathrm {K}$
$\mu$	2

**Table 4:** Thermal properties titanium alloy Ti6Al4V

Thermal conductivity	(24 $^{\circ }\hbox {C}$)	*k*	6.785	N/sK
	(1185 $^{\circ }\hbox {C}$)		24.375	N/sK
Specific heat	(24 $^{\circ }\hbox {C}$)	*c*	502	J/kg K
	(1185 $^{\circ }\hbox {C}$)		756	J/kg K
Expansion coefficient	(100 $^{\circ }\hbox {C}$)	$\alpha$	$10.064\cdot 10^{-6}$	$\hbox {K}^{-1}$
	(1200 $^{\circ }\hbox {C}$)		$12.42\cdot 10^{-6}$	$\hbox {K}^{-1}$

## Thermo-mechanical coupling

The following lines present a summary of the isothermal split, developed in [29]. Let $t_n\rightarrow t_{n+1}$ be the initial and final time step. Let $\Delta t = t_{n+1}-t_n$ be the time increment.

The algorithm in Box 3 is based on the application of an implicit backward-Euler difference scheme to the momentum equation, for fixed initial temperature (temperature at previous time step) and the application of an implicit backward-Euler difference scheme to the energy equation at a fixed configuration (configuration obtained as a solution of the mechanical problem).

The solution of the balance of momentum equation for fixed initial temperature gives an update of the primary variables $\mathbf {u}_{n+1},\,\mathbf {p}_{n+1}$ and a first update of the internal variables (left Cauchy-Green tensor, internal energy and entropy) of the form

23 $$\begin{aligned} \mathbf {b}^e_n, \bar{\mathbf {e}\,}^p_{\,n}, \eta ^{\,p}_n\;\;\rightarrow \;\;(\mathrm {Box}\;~2)\;\;\rightarrow \;\;\tilde{\mathbf {b}}^e_{n+1}, \tilde{{\bar{\mathbf {e}}}}_{\,n+1}, {\tilde{\eta }\,}^{\,p}_{n+1} \end{aligned}$$

Along with an incremental value of the consistency parameter satisfying the *Kuhn–Tucker* conditions and denoted by $\Delta \tilde{\lambda }_{n+1}$.

The solution of the balance of energy with initial conditions $\mathbf {u}_{n+1}, \mathbf {p}_{n+1}, \theta _n$ and initial internal variables $\mathbf {b}^e_n, \bar{\mathbf {e}\,}^p_n, \eta ^{\,p}_n$ gives an update of the primary variable $\theta _{n+1}$ and a second update of the internal plastic variables (at fixed configuration) of the form

24 $$\begin{aligned} \mathbf {b}^e_n, \bar{\mathbf {e}\,}^p_{\,n}, \eta ^{\,p}_n\;\;\rightarrow \;\;(\mathrm {Box}\;~2)\;\;\rightarrow \;\;\tilde{\tilde{\mathbf {b}}}^e_{n+1}, \tilde{\tilde{\bar{\mathbf {e}}}}^p_{n+1}, \tilde{\tilde{\eta }}^{\,p}_{n+1} \end{aligned}$$

Along with an incremental value of the consistency parameter satisfying the *Kuhn–Tucker* conditions and denoted by $\Delta {\tilde{\tilde{\lambda }}}_{n+1}$. In general, $\Delta {\tilde{\lambda }}_{n+1} \ne \Delta {\tilde{\tilde{\lambda }}}_{n+1}$ as a consequence $\tilde{\mathbf {b}}^e_n, \tilde{\bar{\mathbf {e}}}^p_n, \tilde{\eta ^{\,p}}_n\;\ne \;\;\tilde{\tilde{\mathbf {b}}}^e_{n+1}, \tilde{\tilde{\bar{\mathbf {e}}}}^p_{n+1}, \tilde{\tilde{\eta }}^p_{n+1}$.

In summary, the isothermal split solves the mechanical problem with a predicted value of temperature equal to the temperature of the last converged time step and, then, solves the thermal problem using the configuration obtained as a solution of the mechanical problem. A full Newton-Raphson scheme is used for the solution of the non-linear system; the necessary linearization of the constitutive law has been presented in see [21, 29]. The details of the linearization of the weak form of the momentum and energy equation can be seen in [2, 3].

**Table 5:** Implicit isothermal split scheme

1.	Contact Search
	Search for the *Active Set* of Contacts with a Rigid Tool
2.	Mechanical Solution (for a fixed initial temperature)
	(i) Isothermal Elastoplastic problem
	(primary variables $\mathbf {u}_{n+1}, \mathbf {p}_{n+1}$ and internal variables $\mathbf {b}^e_n, \bar{\mathbf {e}\,}^p_{n}, \eta ^p_n$)
	$\bullet$ Iterative Loop
	(a) Implicit integration of the constitutive equation
	$$\mathbf {b}^e_n, \bar{\mathbf {e}\,}^p_{n}, \eta ^p_n\;\rightarrow \;(\mathrm {Box}\; ~2)\;\rightarrow \;\mathbf {b}^e_{n+1}, \bar{\mathbf {e}}^p_{n+1}, {\eta \,}^p_{n+1}$$
	(b) Momentum equation (with the contact constraint)
	$$\mathbf {F}_{\mathbf {u},int}\left( {\varvec{\sigma }}_{n+1}(\mathbf {u}_{n+1},\mathbf {p}_{n+1},\theta _{n};\lambda _{n+1}(\mathbf {u}_{n+1},\theta _{n}))\right) -\mathbf {F}_{\mathbf {u},ext}(\mathbf {u}_{n+1}) = \mathbf {0}$$
	(c) Pressure balance equation (with PPP stabilization )
	$$\mathbf {F}_{\mathbf {p},pres}(\mathbf {p}_{n+1})-\mathbf {F}_{\mathbf {p},vol}(\mathbf {u}_{n+1})+\mathbf {F}_{\mathbf {p},stab}(\mathbf {p}_{n+1})=0$$
	(d) Update the *Active Set* of Contacts
	$\bullet$ Convergence Achieved
	$$\begin{aligned} \mathbf {u}_{n+1}&= \mathbf {u}_{n} + \Delta \mathbf {u}\\ \mathbf {p}_{n+1}&= \mathbf {p}_{n} + \Delta \mathbf {p}_{n+1} \end{aligned}$$
3.	Thermal Solution (for a fixed configuration)
	(ii) Thermoplastic problem
	(primary variable $\theta _{n+1}$ and internal variables $\mathbf {b}^e_n, \bar{\mathbf {e}\,}^p_{n}, \eta ^p_n$)
	$\bullet$ Iterative Loop
	(a) Implicit integration of the constitutive equation
	$$\mathbf {b}^e_n, \bar{\mathbf {e}\,}^p_{n}, \eta ^p_n\;\rightarrow \;(\mathrm {Box}\; ~2)\;\rightarrow \;\mathbf {b}^e_{n+1}, \bar{\mathbf {e}}^p_{n+1}, {\eta \,}^p_{n+1}$$
	(b) Energy equation (with the contact constraint)
	$$\mathbf {F}_{\theta ,dyn}({\dot{\theta }}_{n+1})-\mathbf {F}_{\theta ,int}(\theta _{n+1})+\mathbf {F}_{\theta ,ext}=0$$
	$\bullet$ Convergence Achieved
	$$\theta _{n+1} = \theta _{n} + \dot{\theta }_{n+1} \Delta t$$
The definition of the variables that appear in this box are explained in Sects. 3 and 5 and references [21, 30].	

## The Particle Finite Element Method

The Particle Finite Element Method (PFEM) was developed initially for solving fluid dynamics problems. The method was presented as a novel way to model free surfaces in a Lagrangian manner, following and tracking the fluid particles along the domain (see [11]). The Lagrangian description of the continuum, commonly used in the field of solids, allowed the possibility of the modeling of fluid dynamics and the treatment of the fluid-structure interaction problems in a unified manner. A wide range of simulations has been faced with PFEM since it was first presented: fluid interacting with rigid bodies, used in marine and port engineering; erosion processes, used in river engineering, mixing processes or fluid coupling with thermal effects, industrial granular flows; trying to give a response to many industrial problems [10, 16, 17].

In this paper we apply the method to the modeling of industrial metal cutting processes, concentrating our efforts on the modeling of segmental chip formation in the cut. The Lagrangian description of the continuum is something common within the theory of solids for the Finite Element Method. However finite elements have some critical limitations when they deform too much and its shape is distorted. That occurs when the material suffers large strains as is the case of the concerning problem. The role of the particle description of domain is to introduce a solution for this matter.

The original idea of the PFEM was to improve the mesh quality by performing a re-tessellation of the domain. This must be used only when is needed and localized to the regions with critical distortion. That allows to adapt to the large changes of the meshing domain and avoid global remeshing and interpolation from mesh to mesh at each computing step. In 2D, the re-tessellation consists in re-computing the element connectivity using a Delaunay triangulation [6, 26] where the current position of the particles (i.e., of the mesh nodes) is kept fixed. Mesh distortion is corrected and improved naturally with the PFEM, because the triangulation maximize the minimum angle of all the angles of the triangles of the new mesh. Therefore it tends to avoid distorted skinny triangles.

First applications of the PFEM to solid mechanics were done in problems involving large strains, large rotations, multi-body contacts and creation of new surfaces (riveting, powder filling and machining,shearing) [14, 19, 20, 22–24]. In this work, we extended the Particle Finite Element Method to the numerical simulation of metal cutting processes involving the transition from continuous to serrated chip with the increment of the cutting speed.

**Fig. 4:**
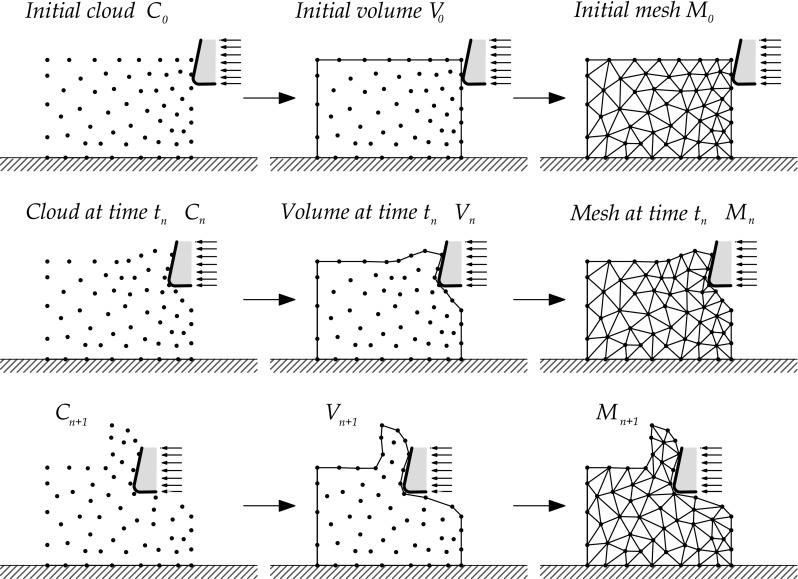
Remeshing steps in a standard PFEM numerical simulation

### Basic steps of the PFEM

The PFEM is characterized by the particle description of the material domain. A cloud of material particles are considered to define the continuum domain. A Lagrangian description of the analysis domain is used to define the particles motion which is tracked during the transient solution. An incremental update of the body configuration is done at each time step with the use of the Updated Lagrangian formulation. The continuum equations are solved with the FEM. Hence a mesh of FE must be generated with the re-connection of the material particles discretizing the continuum. The quality of the numerical solution improves with the discretization, so the number and the location of the material particles is going to improve the problem resolution. Adaptive mesh refinement techniques will be used to improve the resultant particle distribution.

For clarity purposes the steps of typical solution with the PFEM are described next. The starting point is a collection or cloud of nodes (C) pertaining to the analysis domain. The volume (V) of the analysis domain is defined by the contours of that cloud of nodes. A mesh (M) discretizing the domain is obtained by the re-connection of the nodes. An example of the remeshing scheme using PFEM is shown in Fig. 4. The solution involves the following steps:

1. Definition of the domain(s) ${{\Omega }_{n}}$ in the last converged configuration, $t=t_n$, keeping existing spatial discretization ${{\bar{\Omega }}_{n}}$.
2. Discretization of the given domain(s) in a set of particles of infinitesimal size elimination of existing connectivities ${{\bar{\Omega }}_{n}}$.
3. Reconstruction of the mesh through a triangulation of the domains convex-hull and the definition of the boundary applying the $\alpha$-shape method [9], defining a new spatial discretization.
4. A contact method to recognize the multibody interaction.
5. Solution of the system of equations for $t_{n+1}=t_{n}+\Delta t$.
6. Go back to step 1 and repeat the solution process for the next time step.

The transference of elemental variables that is a critical aspect in solids but less in fluids is usually performed by a smoothing process. This process consists in transfer the information of Gauss points to nodes (particles) before the elimination of the existing elements connectivity (step 2) and recover the information with the interpolation of the nodal variables into Gauss points before to perform the solution of the system equations (step 5). The improved transfer of elemental information used in this work is going to be described in detail in Sect. 7.2.

Although we are not going to make use of this aspect in our modeling, highlight that with this description is easy to model separation of particles from the main domain such as fracture of the chip in metal cutting problems. In the case of individual particles segregation, their isolated motion is determined taking into account that a particle has a known density, a known initial acceleration and velocity, and is subjected to gravity forces.

**Fig. 5:**
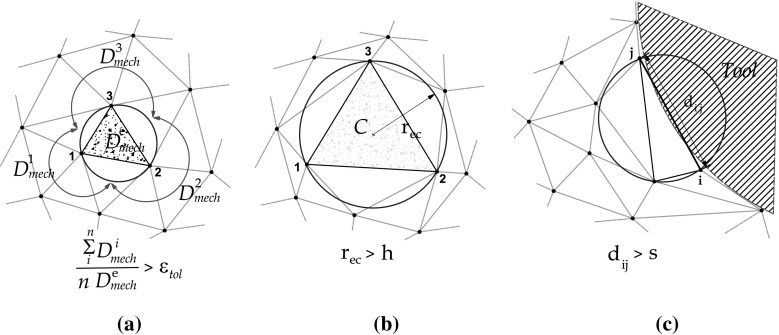
Three main criteria to add a new particle

### Meshing procedure and variables transfer in the PFEM

The meshing procedure is a process of crucial importance in a typical solution with the PFEM. The continuous remeshing is performed via Delaunay triangulation which build a discretized domain with certain particular properties. The Delaunay tessellation generates the convex figure of minimum area that encloses all the points of the domain. The resultant mesh may be not conformal with the external boundaries of the solid domain. If the external boundaries are not preserved the conservation of volume of the domain is not necessarily fulfilled. This is a relevant weakness of the method which is not a valid solution for solid mechanics problems.

In fluids, the tracking of the boundaries (free surfaces) is a part of the method capabilities and the boundary recognition problem is overcome with the so-called $\alpha$-shape method [9]. With the $\alpha$-shape method one can consider that an element of the convex hull conforms an external boundary if fulfills a certain characteristic mesh size. That solves the problem partially, because the resultant external surface generated using $\alpha$-shapes will be different from the previous known external boundaries. There also exist other techniques that combine the $\alpha$-shapes with the previous boundary magnitudes, like normal vectors and directions, that used with the proper algorithm also preserve the contour surface and the volume of the model [5]. Note that in order to deal with complex material flows, where the material can be merged, the use of $\alpha$-shapes is essential.

**Fig. 6:**
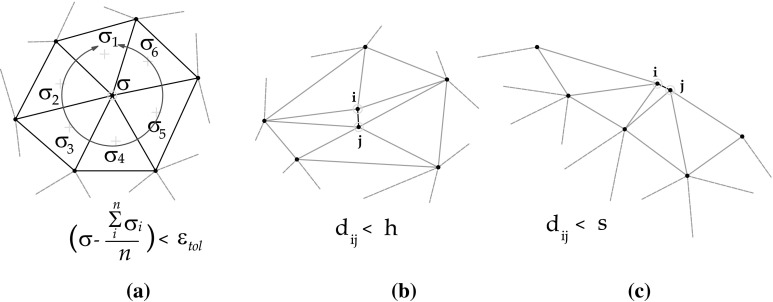
Three main criteria to remove a particle

**Fig. 7:**
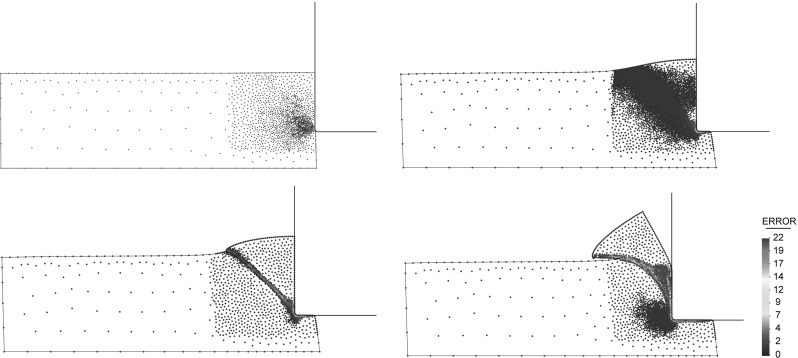
Sequence of refinement for a simulation of linear cutting test. Percentage of the mean error on the equivalent plastic strain value depicted on particles

In this work the $\alpha$-shape method has been replaced by an alternative. In order to preserve exactly the domain contour, a constrained Delaunay algorithm [6, 26] is used. If boundaries are constrained the external boundaries are preserved and the volume conservation is guaranteed. In Delaunay triangulations the constrained tessellation is not difficult to achieve, however in certain non-convex 3D domains the constrained tetrahedralization is not always possible. In these cases, one possible solution consists in adding some nodes on the border of the domain. This is not a big deal for the PFEM, where the addition and removal of particles will be part of the meshing procedure. Applying these techniques, the constrained algorithm allows to perform 2D and 3D modeling. In this work we will focus in the analysis of 2D models.

#### Adding and removing particles

In the Lagrangian approach, the particles move because of the material flow and it may happen that particles concentrate in some regions of the domain and, on the contrary, in other regions the number of particles becomes too low to obtain an accurate solution. To overcome these difficulties, basic PFEM adds and removes particles comparing with a certain characteristic distance.

The addition and removal of particles is one of the principal tools that we have improved and extended in this work in order to model properly the segmental creation of the chips in a machining problem. By inserting and removing particles, the main difficulties associated with element distortion can be solved. The insertion of particles also allows for the resolution of the different scales of the solution. That will permit to capture the plastic shear bands that occur during the cut of a metal-like material.

The insertion of particles we propose applies geometrical and mechanical criteria. The critical cases for the addition of new particles are depicted in Fig. 5. The geometrical criteria are based in a characteristic mesh sizes, i.e. *h* for the element size, *s* for the boundary. The mechanical criterion is based in the equal distribution of the plastic power, such that, elements exceeding the prescribed tolerance ${\varepsilon }_{tol}$ are targeted for refinement.

25 $$\begin{aligned} \int _{\Omega ^{e}}{Q\,d\,{\Omega ^{e}} } > {\varepsilon }_{tol} \end{aligned}$$

where *Q* is the mechanical power (see [27, 28] ) and ${\Omega ^{e}}$ is the domain of the element. When the condition is fulfilled, a particle is inserted in the Gauss point of the finite element.

The most general geometrical insertion criterion is based in the radius of an element circumsphere ${r}_{\mathrm {ec}}$, when ${r}_{\mathrm {ec}}>h$, a new node is added at the center of the circumsphere or at one of the sides of the element. For the boundary, another characteristic distance *s* is defined, when the distance between boundary particles ${d}_{ij}>s$, a new node is added in the center of the boundary segment. The characteristic distance for the boundary *s* has a direct relationship with the geometrical resolution of the tool tip, where the cut of the material is originated. The insertion of particles in the boundary is a fundamental process in metal cutting problems. The geometrical description of the cut is done by the insertion of particles in the boundary of the workpiece at the tool tip. This allows for the contour increase and creates the shape of the cut.

Similar criteria are defined for the removal of particles. The critical cases for the removal of particles are depicted in Fig. 6. If the distance between two nodes ${d}_{ij}<h$ is smaller than certain characteristic distance *h*, one of the nodes will removed. A particular criterion with a different characteristic distance *s* is applied for the boundary segments, if ${d}_{ij}<s$, one of the nodes will removed.

The mechanical criterion for removing particles is based on error estimators, a particle is removed if the error $\Vert \sigma -\sigma _{h}\Vert <{\varepsilon }_{tol}$ is smaller that a given tolerance ${\varepsilon }_{tol}$. The used error estimators are based on plastic strain values or on the norm of the isochoric-stress. These two magnitudes use to describe the region of interest where the different scale of the physical solution is taking place. The absence of error on them says that nothing relevant occur in that zone and therefore the finite element mesh can be coarsened if needed.

**Table 6:** Flowchart of the refining scheme and information transfer process for the PFEM

Mesh Refinement scheme for the PFEM	
1.	Update the particle positions: $\;\;\;\;\;\;\;\mathbf {x}_{n+1} = \mathbf {x}_{n} + \Delta \mathbf {u}$
2.	Remove Particles.
	Criterion based on distances and error estimators. Figure 6.
3.	Refine boundary that is too distorted
	Criterion based on distance. Figure 5 (c).
4.	Perform a Constrained Delaunay triangulation.
	The triangulation must include remaining particles and preserve boundaries.
5.	Refine Elements adding new particles.
	Criterion based error estimators and distances. Figure 5, (a) and (b).
6.	Refine previous Delaunay triangulation.
	The new triangulation must include new particles and preserve the boundaries.
Information Transfer scheme for the PFEM	
1.	Calculate the integration points global coordinates of the of the new triangulation.
2.	Update the internal variables of the new triangulation.
	This step states that the integration point information of finite element in the new mesh corresponds to the integration point information of the closest finite element in the previous mesh. The information of the previous mesh is needed to perform this transfer.
It is important to remark that step 2 is optional.	
The main advantage of the proposed strategy is that:	
It is not necessary to create a complete new mesh (without step 2); we only adapt the mesh with the addition of new particles and the mesh quality is improved using a *Delaunay* triangulation insertion algorithm.	

The refinement can be applied globally to all domain or only in a certain predefined zone of interest. Note that the zone of interest, in a problem of cutting, can be defined as a spatial bounding box close to the cutting tool. The refinement procedure applied to a numerical modeling of a linear cutting test is presented in Fig. 7.

#### Elemental variables transfer

In the PFEM, particles preserve the information from mesh to mesh. All the information necessary in subsequent time steps has to be kept, the particle information includes nodal displacements, temperatures, pressures, geometrical domain labels, and characteristic sizes. If the number of particles is not modified the information is automatically preserved. Only in the case of the inserted particles an interpolation is needed to define their new nodal information. In the case of removed particles, the information stored in them is lost for the subsequent time steps, but it is kept until the end of the meshing procedure to perform the interpolations with fidelity.

The coarsening of the mesh reduces the computational cost of the analysis, however some information is lost during this process. If we are interested in the analysis of the residual stresses the coarsening must be avoided.

As was explained in Sect. 7.1, the transfer of elemental information in a typical simulation with the PFEM lead with certain smoothing of the transferred variables. That was because the elemental information is transferred to nodes and interpolated again to the new elements when the new mesh is performed. This transfer scheme is in line with the particle description of the continuum. However the loss of information due to that transfer is severe and is a very diffusive process, even if you transfer only the increments of the elemental values.

If we look at the places where the mesh does not change, using this transfer procedure the solution is smoothed anyway and the equilibrium after meshing is lost. Nevertheless, this not happens if the transfer is done directly from the previous element to the new element. When the information is transferred between elements, if the mesh not changes, the solution is the same and the equilibrium is preserved. When the mesh is not the same, the equilibrium is perturbed only in areas where large changes occurred and the transformation of the information is unavoidable.

**Fig. 8:**
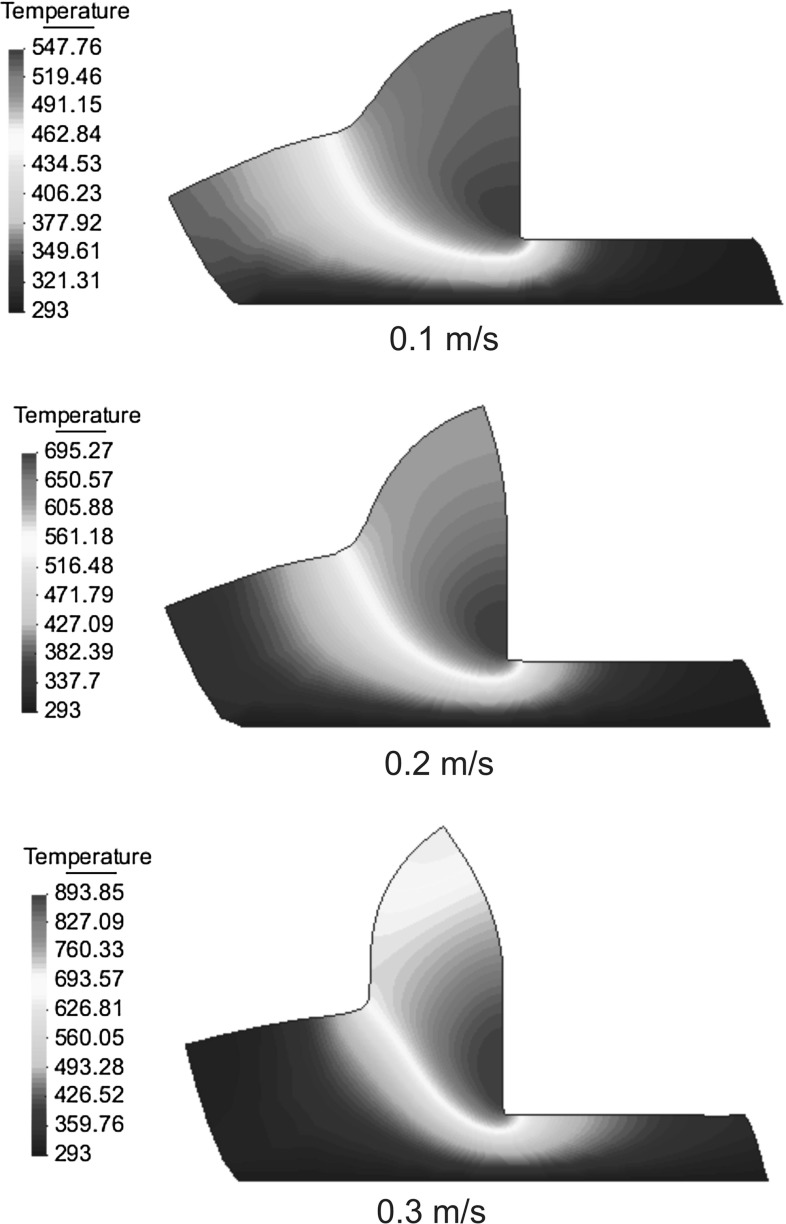
Comparison of chip morphologies of Ti6Al4V at different cutting speeds (temperature in Kelvins (K) )

**Fig. 9:**
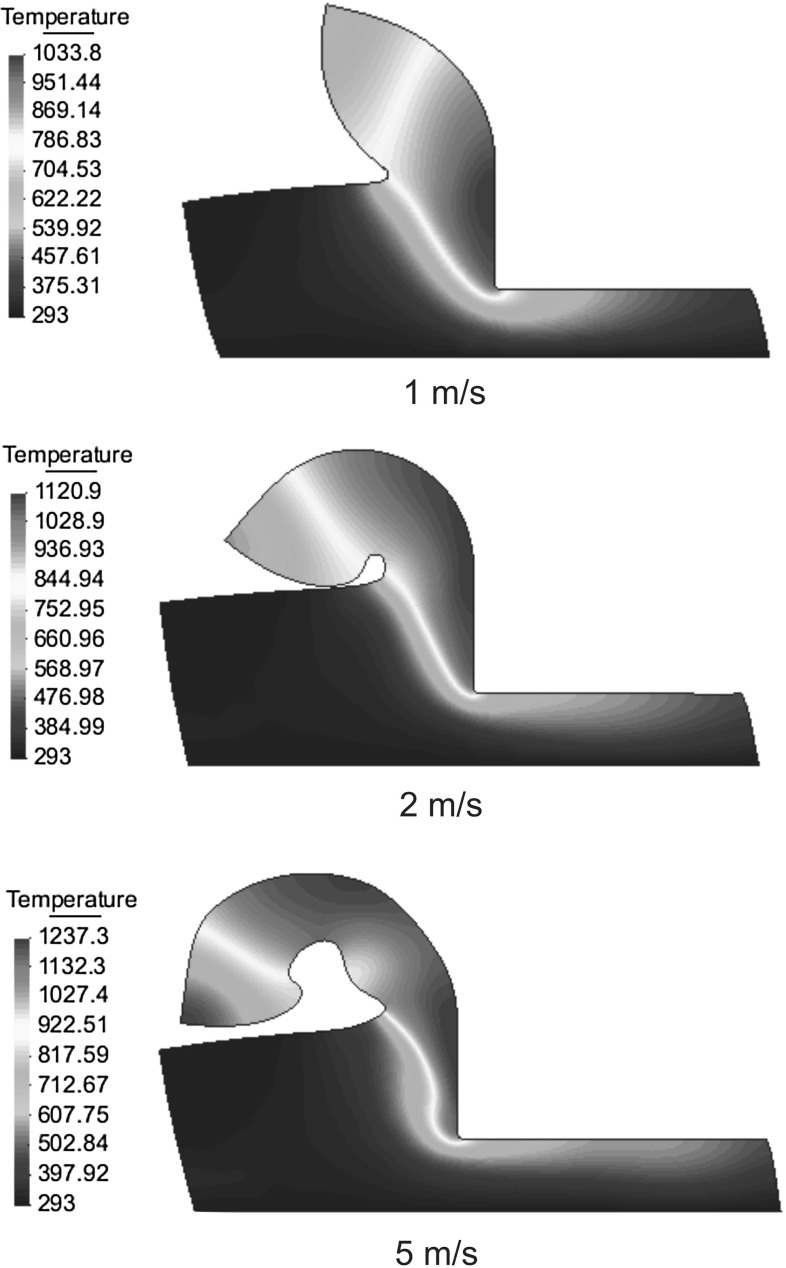
Comparison of chip morphologies of Ti6Al4V at different cutting speeds (temperature in Kelvins (K) )

In Box 4, the procedure for mesh refinement and information transfer is shown. This procedure is the one that is applied in the examples presented in this paper for the simulation of segmental chip formation in metal cutting processes (see Fig. 7).

## Examples

### Machining a titanium alloy (Ti6Al4V) at different cutting speeds. The effect on cutting forces and chip shapes

**Fig. 10:**
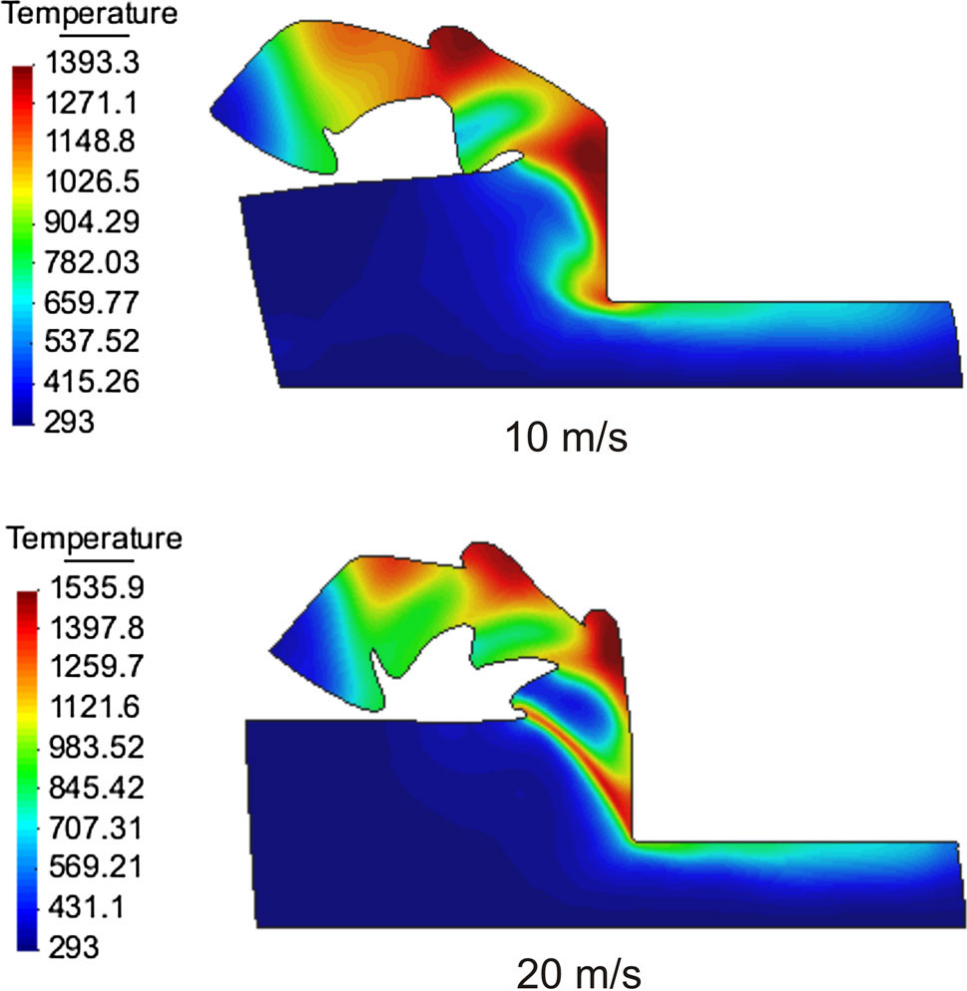
Comparison of chip morphologies of Ti6Al4V at different cutting speeds (temperature in Kelvins (K) )

The application concerns the cutting of a rectangular block of Ti6Al4V alloy of length 200 $\upmu \hbox {m}$ and width 60 $\upmu \hbox {m}$, a cutting depth of 35 $\upmu \hbox {m}$, a rake angle of 0 and a tool radius of 2 $\upmu \hbox {m}$. The cutting speed has been varied between 0.1 and 20 m/s. Material behavior is given by a modified Johnson–Cook law (Bäker law) with the materials properties shown in Tables 1 and 2. The conductivity and specific heat depend linearly on the temperature. The tool has been assumed to be mechanically rigid, the friction and the thermal exchange between the work piece and the tool are neglected. The solution scheme used in the present example is based on the isothermal implicit scheme and the remeshing is based on the particle finite element method (PFEM) presented in Sects. 7 and 6. Insertion and removal of particles is used in this example to save computing time and in order to improve the localization phenomenon. Material separation in front of the tool has been modeled by considering the chip formation process as a pure deformation where material flows visco-plastically around the tool tip. An additional tool is used in order to avoid chip penetration in the workpiece. A total of 4000 time steps were needed in order to calculate any of the chips shown in Figs. 8, 9 and 10, the standard computing time was 2.5–9 h on a computer running with the processor Intel Core 2 Duo @ 2.53 Ghz. The example presented in this section has been taken from Bäker [1] and De Micheli [7]. An especially appealing feature of high-speed cutting processes is that the specific cutting force for most materials strongly decreases with increasing the cutting speed. The frequently observed transition between continuous and segmented chip is reproduced by the model. Figures 8, 9, and 10 show the temperature field for seven different values of cutting speeds. At small cutting speed, continuous chip are formed with increasing the shear angle. Chip segmentation is observed at cutting speeds higher than 5 m/s and the segmentation increases with the increase of the cutting speed.

Figures 8, 9, and 10 show another interesting phenomenon: the width of the shear zone in the continuous chip becomes smaller with the increase of the cutting speed. As the increasing temperature causes a decrease in hardening, the width of the shear zone becomes smaller so that strain rates becomes larger.

#### The cutting forces

**Fig. 11:**
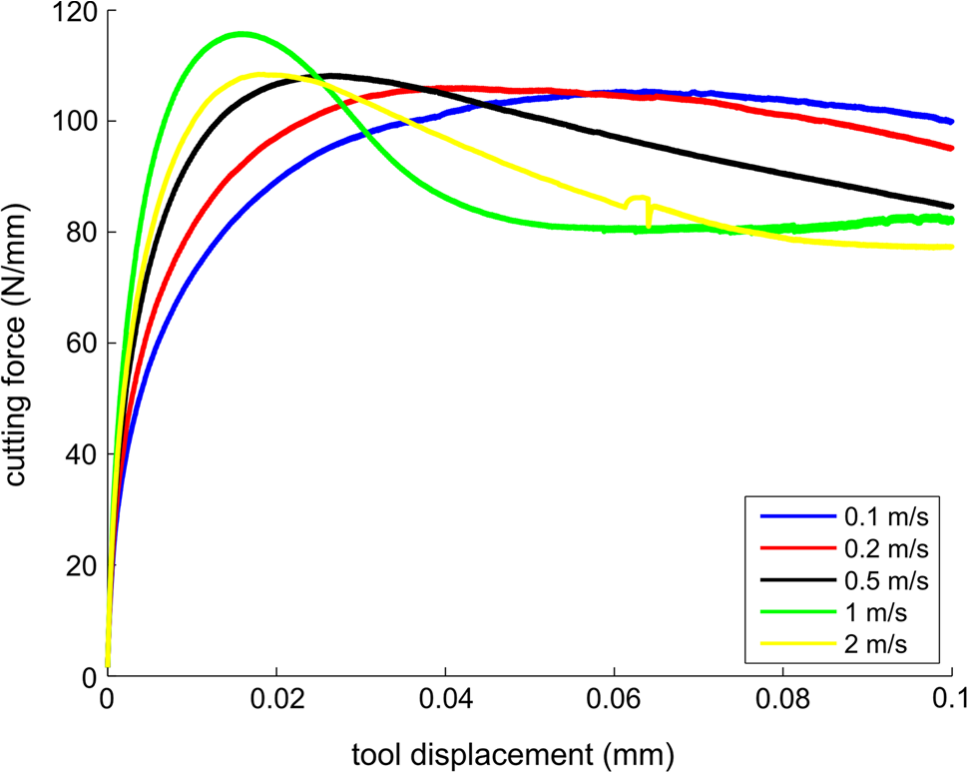
Cutting forces of Ti6Al4V at different cutting speeds

**Fig. 12:**
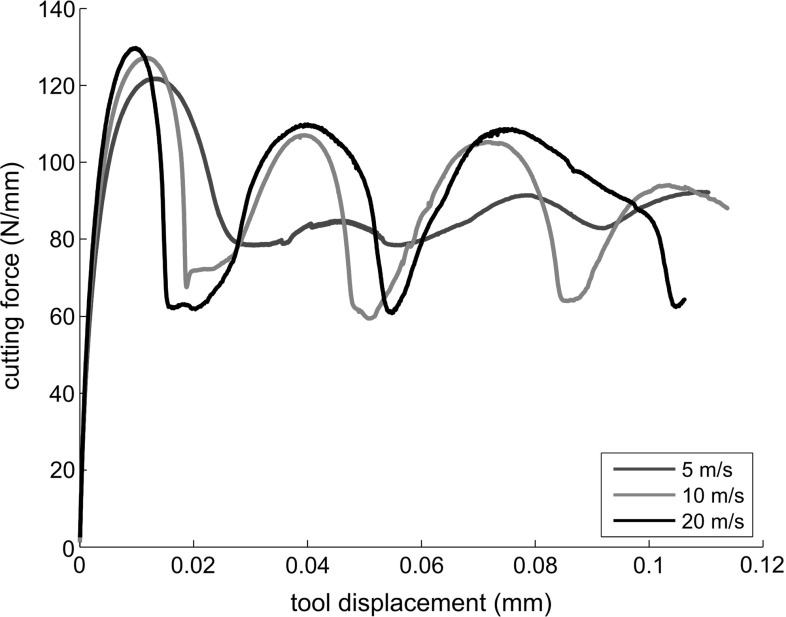
Cutting forces of Ti6Al4V at different cutting speeds

Plots of the cutting force are shown in Figs. 11 and 12. The plots are distance-resolved, in such a way that results for different cutting speeds are comparable. For the continuous chip formation, the cutting force tends to a constant value, whereas, for the segmental chip formation, the cutting force oscillates around a mean value. The observed decrease in the cutting force at high cutting speed can be thus explained as follows: increasing the cutting speed causes an increase in the temperature. Although the strain rate increment, causes a larger isothermal flow stress, the increment of temperature leads to thermal softening, so that the mean flow stress is reduced. In conclusion, the simulations show that the large decrement of the cutting force associated with the increment of the cutting speed is mainly a result of the thermal softening which changes the effective stress-strain curve and increases the shear angle.

**Fig. 13:**
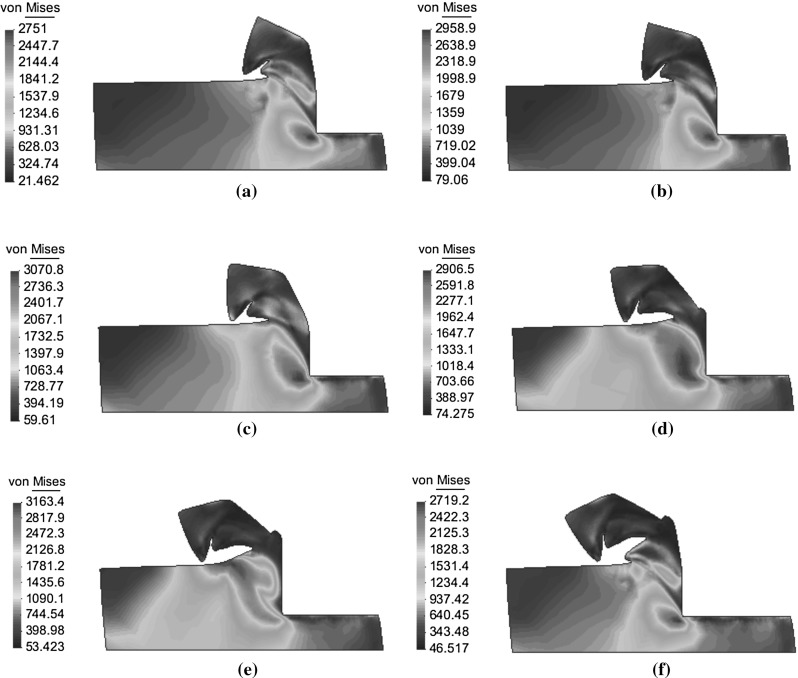
Development of the von Mises stress inside a chip for a cutting speed of 20 m/s (MPa)

#### Adiabatic shear band formation process

In Fig. 13, we can observe in detail the chip formation of an adiabatic shear band in our simulation at 20 m/s. The evolution of the cut is shown in six different time instants, going from Fig. 13a–f, in all of them the contour fill for the *von Mises* stress is depicted. The instant represented by the Fig. 13a shows a state where one shear band is nearly fully developed and the deformation occurs mainly along this strongly curved band. In the instant depicted by the Fig. 13b the deformation occurs in this band, but there is also some deformation in the region behind this shear band, leading to a damming of the material. Generally, the concentration of the deformation begins at the tool tip, but a second deformation concentration starts at the free surface, close to the tool rake face (see Fig. 13c) before the shear band is fully formed. The cutting force increases during the first stages and reaches a maximum during this phase because the newly forming shear band. Although the deformation concentrates during the instants shown in Fig. 13c, d, this shear localization is not coloured by the plot of the *von Mises* stress contour fill. During this cutting fase, the *von Mises* stress inside the zone where deformation concentrates is larger than in the adjacent zones, mainly due to the higher strain rates in this region. In the instance depicted in Fig. 13e, true strain localization has begun near the tool tip as the *von Mises* stress experience a important decrement there. This has a direct correlation with the large increment in the temperature field. At that instant, the area of deformation concentrates at the free surface, which is not a localized deformed area because the *von Mises* stress is larger there than in the adjacent zones.

Therefore there are two zones where the deformation concentrates, the main localization of the deformation grows continuously from the tool tip and afterwards the two zones join. When the localization has started; no further deformation takes place in the dammed region behind the shear band. When the two areas of large deformation are joined all strains get concentrated inside the shear band (see Fig. 13f).

## Conclusions

In this work, the chip formation of Ti6Al4V during cutting is studied using revised Particle Finite Element Method. A first conclusion is that PFEM is well adapted for the simulation of the complex processes involved. The obtained results are in good agreement with those presented in the literature (see [1, 7]).

Effects observed experimentally for many materials, namely a reduction in the cutting force followed by a plateau region, and the transition between continuous and segmented chips, have been successfully reproduced by the PFEM simulation. It was shown that the reduction in the cutting force can be understood as an effect of thermal softening which causes a change in the effective stress-strain curves of the material and thus increases the shear angle and reduces the amount of plastic deformation necessary to deform the chip. The transition from continuous to segmented chips causes a further reduction in the cutting force, which is, however, much smaller. The results presented in this paper have thus given evidence that experimentally observed trends in chip formation can be predicted by PFEM.

In conclusion, it should be noted that investigating an idealized process (neglection of friction, deformation of the tool and a simplified flow stress law) seems to be a fruitful method to understand details of the chip formation process. A necessary improvement of the model presented in this paper concerns in the inclusion of the neglected phenomena (friction, deformation of the tool, more complex flow stress and the heat transfer between the tool and the workpiece) (see [20]).

## References

[1] Bäker M (2006). Finite element simulation of high-speed cutting forces. J Mater Process Technol.

[2] Belytschko T, Liu WK, Moran B (2000). Nonlinear finite element for continua and structures.

[3] Bonet J, Wood RD (1997). Nonlinear continuum mechanics for finite element analysis.

[4] Cante J, Dávalos C, Hernández JA, Oliver J, Jonsén P, Gustafsson G, Häggblad HÄ (2014). PFEM-based modeling of industrial granular flows. Computat Part Mech.

[5] Carbonell JM (2009) Modeling of ground excavation with the particle finite element method. Ph.D. thesis, Universitat Politècnica de Catalunya (UPC), Barcelona

[6] Delaunay BN (1934). Sur la Sphère Vide, A la memoire de Georges Voronoi. Otdelenie Matematicheskii i Estestvennyka Nauk.

[7] De Micheli PO, Mocellin K (2011). 2D high speed machining simulations using a new explicit formulation with linear triangular elements. Int Mach Mach Mater.

[8] Dohrmann CR, Bochev PB (2004). A stabilized finite element method for the Stokes problem based on polynomial pressure projections. Int J Numer Methods Fluids.

[9] Edelsbrunner H, Mucke EP (1994). Three dimensional alpha shapes. ACM Trans Graph.

[10] Franci A (2015) Unified Lagrangian formulation for fluid and solid mechanics, fluid-structure interaction and coupled thermal problems using the PFEM. Ph.D. thesis, Universitat Politècnica de Catalunya (UPC), Barcelona

[11] Idelsohn SR, Oñate E, Pin FD (2004). The particle finite element method: a powerful tool to solve incompressible flows with free-surfaces and breaking waves. Int J Numer Methods Eng.

[12] Marusich TD, Ortiz M (1995). Modelling and simulation of high-speed machining. Int J Numer Methods Eng.

[13] Moran B, Ortiz M, Shih CF (1990). Formulation of implicit finite element methods for multiplicative finite deformation plasticity. Int J Numer Methods Eng.

[14] Oliver J, Cante JC, Weyler R, González C, Hernández J (2007). Particle finite element methods in solid mechanics problems. Computat Methods Appl Sci.

[15] Oliver J, Huespe AE, Cante JC (2008). An implicit/explicit integration scheme to increase computability of non-linear material and contact/friction problems. Comput Methods Appl Mech Eng.

[16] Oñate E, Celigueta MA, Idelsohn SR (2006). Modeling bed erosion in free surface flows by the particle finite element method. Acta Geotech.

[17] Oñate E, Idelsohn SR, Celigueta MA, Rossi R (2008). Advances in the particle finite element method for the analysis of fluid-multibody interaction and bed erosion in free surface flows. Comput Methods Appl Mech Eng.

[18] Owen DRJ, Vaz M (1999). Computational techniques applied to high-speed machining under adiabatic strain localization conditions. Comput Methods Appl Mech Eng.

[19] Rodríguez JM (2014) Numerical modeling of metal cutting processes using the particle finite element method(PFEM). Ph.D. thesis, Universitat Politècnica de Catalunya (UPC), Barcelona

[20] Rodríguez JM, Cante JC, Oliver J (2015). On the numerical modelling of machining processes via the Particle finite Element method (PFEM). CIMNE: Barcelona.

[21] Rodríguez JM, Carbonell JM, Cante JC, Oliver J (2016). The particle finite element method (PFEM) in thermo mechanical problems. Int J Numer Methods Eng.

[22] Rodriguez JM, Jonsén P, Svoboda A (2016). Simulation of metal cutting using the particle finite-element method and a physically based plasticity model. Comput Part Mech.

[23] Sabel M, Sator C, Müller R (2014). A particle finite element method for machining simulations. Comput Mech.

[24] Sabel M, Sator C, Zohdi TI, Müller R (2016). Application of the particle finite element method in machining simulation discussion of the alpha-shape method in the context of strength of materials. ASME J Comput Inf Sci Eng.

[25] Sekhon GS, Chenot JL (1993). Numerical simulation of continuous chip formation during non-steady orthogonal cutting simulation. Eng Comput.

[26] Shewchuk JR (1998) A condition guaranteeing the existence of higher-dimensional constrained Delaunay triangulations. In: Proceedings of the fourteenth annual symposium on Computational geometry, ACM, Minneapolis, MN, USA, pp 76–85

[27] Simo JC (1988). A framework for finite strain elastoplasticity based on maximum plastic dissipation and the multiplicative decomposition: part I. Continuum formulation. Comput Methods Appl Mech Eng.

[28] Simo JC (1988). A framework for finite strain elastoplasticity based on maximum plastic dissipation and the multiplicative decomposition. Part II: computational aspects. Comput Methods Appl Mech Eng.

[29] Simo JC, Miehe C (1992). Associative coupled thermoplasticity at finite strains: Formulation, numerical analysis and implementation. Comput Methods Appl Mech Eng.

[30] Simo JC, Hughes TJR (1998). Computational inelasticity.

[31] Strenkowski JS, Carroll JT (1985). A finite element model of orthogonal metal cutting. J Eng Ind Trans ASME.

[32] Zhang X, Krabbenhoft K, Sheng D, Li W (2015). Numerical simulation of a flow-like landslide using the particle finite element method. Comput Mech.

